# Homo- and Heterogeneous Glycoconjugates on the Basis of *N*-Glycans and Human Serum Albumin: Synthesis and Biological Evaluation

**DOI:** 10.3390/molecules27041285

**Published:** 2022-02-14

**Authors:** Almira Kurbangalieva, Regina Zamalieva, Igor Nasibullin, Kenshiro Yamada, Katsunori Tanaka

**Affiliations:** 1 Biofunctional Chemistry Laboratory, A. Butlerov Institute of Chemistry, Kazan Federal University, 18 Kremlyovskaya Street, 420008 Kazan, Russia; regina.sibgatullina@mail.ru (R.Z.); igor.nasibullin@riken.jp (I.N.); 2 Biofunctional Synthetic Chemistry Laboratory, RIKEN Cluster for Pioneering Research, 2-1 Hirosawa, Wako-shi, Saitama 351-0198, Japan; 3 Department of Chemical Science and Engineering, School of Materials and Chemical Technology, Tokyo Institute of Technology, 2-12-1 O-okayama, Meguro-ku, Tokyo 152-8552, Japan; yamada.k.bv@m.titech.ac.jp

**Keywords:** *N*-glycans, albumin, glycocluster, glycoconjugation, biodistribution, pattern recognition, in vivo molecular imaging, tumor targeting, drug delivery system, chemical biology

## Abstract

Neoglycoconjugates mimicking natural compounds and possessing a variety of biological functions are very successful tools for researchers to understand the general mechanisms of many biological processes in living organisms. These substances are characterized by high biotolerance and specificity, with low toxicity. Due to the difficult isolation of individual glycoclusters from biological objects, special interest has been directed toward synthetic analogs. This review is mainly focused on the one-pot, double-click methodology (containing alkyne–azide click cycloaddition with the following 6π-azaelectrocyclization reactions) used in the synthesis of *N*-glycoconjugates. Homogeneous (including one type of biantennary *N*-glycan fragments) and heterogeneous (containing two to four types of biantennary *N*-glycan fragments) glycoclusters on albumin were synthesized via this strategy. A series of cell-, tissue- and animal-based experiments proved glycoclusters to be a very promising class of targeted delivery systems. Depending on the oligosaccharide units combined in the cluster, their amount, and arrangement relative to one another, conjugates can recognize various cells, including cancer cells, with high selectivity. These results open new perspectives for affected tissue visualization and treatment.

## 1. Introduction

Cells are striking in their diversity within organisms. However, in all this variety, there is at least one thing in common—sugars. Without any exception, all cells and numerous macromolecules in nature carry an array of covalently attached mono- or oligosaccharides, which are generically referred to as “glycans”. Located on the outer surface of cellular and secreted macromolecules, glycans are involved in various processes of living systems, such as fertilization, cell growth, differentiation, host–pathogen communication, etc. [1,2,3,4].

In medicine, natural glycoproteins are indispensable for the prevention and treatment of various diseases, due to their high specificity, low toxicity, and defined biological functions [5,6,7]. However, because of the nature of biological macromolecules, they also have several disadvantages. One of the problems is the complex composition and structure of carbohydrate units.

There are two common ways to link glycan and protein. A feature in the case of *N*-linked glycans is their binding to the nitrogen atom of asparagine. Meanwhile, *O*-linking occurs to the oxygen atom of serine or threonine [1,2,8]. According to the literature, exactly *N*-glycoproteins are involved in intercellular recognition due to highly specific lectin–carbohydrate interactions. The specificity of interaction depends on both the peptide component of the molecule and the structure of the carbohydrate chain [1,2,3,4,9].

It is well known that cell–cell interaction based on pattern recognition is very specific and works as a lock-and-key system. However, a molecular basis of pattern recognition still remains largely unknown [10]. In this context, imitation of natural glycan pattern recognition by a recreation of homogeneous and heterogeneous neoglycoconjugates could provide more knowledge about glycans functions and trimming-dependent interaction mechanisms. Moreover, this could be also used in the development of breakthrough diagnostic and therapeutic *N*-glycoconjugate tracers [11,12,13].

Nowadays, a large number of chemical and enzymatic approaches have been developed for the synthesis of glycoclusters. The use of various platforms in combination with the existing synthetic methods potentially allows obtaining conjugates that contain 1–10 glycan fragments, preprogramming their arrangement, introducing both mono- and oligosaccharide residues, obtaining homo- or heterogeneous glycoconjugates, varying the spacer between oligosaccharide and biotemplate, etc. [14,15].

At the same time, not all clusters can be useful in terms of biological research. Studies of various multivalent synthetic glycan-linked biomolecules (so-called neoglycoconjugates) obtained on the basis of dendrimers, nanoparticles, liposomes, etc. broadly fall into two types with separate focuses [13,16,17,18,19,20,21]. The comprehensive in vitro analysis of glycoclusters is the main topic of the first field [22,23]. Another research field utilizes cell- and animal-based studies.

Serum proteins, such as albumin, are the ideal delivery templates of choice by many scientific groups. Due to their natural occurrence, availability of many reactive groups for glycan clustering, in vivo stability, and negligible immunogenicity in clinical applications, synthetic glycoalbumins should experience little biological interference in the blood [24].

Considering all the advantages of albumins many researchers are concentrated on the synthetic methods development and biological study of protein-based glycoconjugates. The most popular proteins to be used are human serum albumin (HSA) and bovine serum albumin (BSA). We will not discuss the synthesis or isolation of glycan fragments here but mention only some examples of conjugation of glycans with albumins and biological evaluation of clusters.

The most common is the conjugation with amino or thiol groups of proteins. Conjugation with the SH group of cysteine allows for the effective attainment of mono- or disubstituted glycoproteins due to the low natural content of cysteine [11,25,26,27]. Conjugation with NH_2_ groups is superior in terms of obtaining multivalent glycoclusters. Among the various methods using acylation, reductive amination, reactions with squaric acid esters, isothiocyanates, isocyanates, acyl halides, azides, imidoesters, anhydrides, carbonate compounds [28,29,30,31,32,33,34,35,36,37,38], the so-called click-reaction methods are the most beneficial.

**Figure 1:**
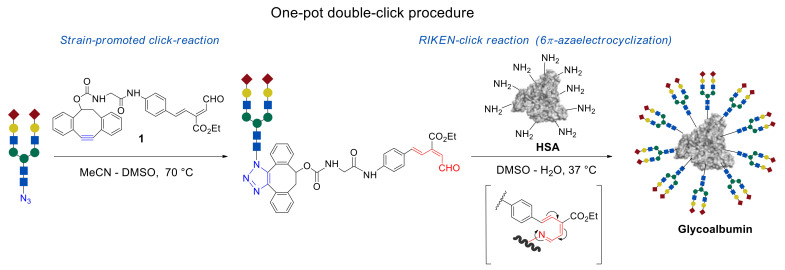
A strategy of two sequential click reactions based on a strain-promoted, click reaction of the dibenzocyclooctyne (azide–alkyne cycloaddition), followed by a 6π-azaelectrocyclization of unsaturated imines in the synthesis of glycoalbumins [43].

In this review, we focus on the one-pot, double-click strategy developed and improved by Dr. Katsunori Tanaka et al. This procedure is based on strain-promoted click reaction of dibenzocyclooctyne (alkyne–azide click cycloaddition) [38] with the subsequent 6π-azaelectrocyclization—RIKEN click reaction (Figure 1) [11,39,40,41,42]. The usage of compound **1**, carrying a strained C ≡ C bond and unsaturated aldehyde fragment allows glycans immobilization onto the NH_2_ group containing templates by the first click reaction of strained alkyne with azide group of *N*-glycan molecules, followed by the second RIKEN click reaction of unsaturated aldehyde group with lysine residue of HSA. The advantages of this methodology, in comparison with the conventional organic reactions, are its efficiency and selectivity, mild reaction conditions, and rate of reaction.

This review also considers recent achievements of in vivo kinetics and tumor-targeting studies with the use of HSA-based neoglycoconjugates. There is remarkable dependence of in vivo kinetics and organ-specific accumulation of glycoclusters on the use of HSA-based neoglycoconjugates, as verified by biodistribution and imaging studies.

## 2. Homogeneous Glycoalbumins

**Figure 2:**
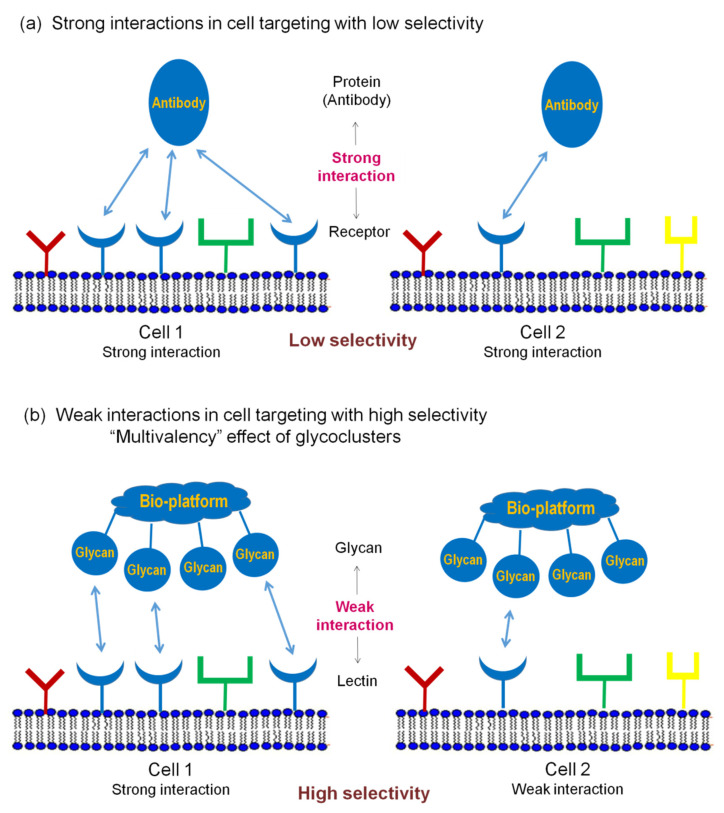
Cells targeting using (**a**) strong interactions, (**b**) weak interactions enhanced with multivalency effect of glycoconjugates. Receptors and glycans/antibodies of the same shape and color have interaction with each other. Bio-platform: cell, antibody, protein, etc. [44]. Copyright Wiley-VCH GmbH. Reproduced with permission.

Glycan multivalency effect is an important aspect of the pattern recognition mechanisms. The pattern recognition concept, using glycans for strong interactions with target cells, is proposed in Figure 2.

Each type of cell can be characterized by a specific combination of surface receptors. Some receptors can be the same for different cells (e.g., blue, round-shaped receptors on cells 1 and 2 in Figure 2). Molecules such as antibodies, which interact strongly with antigens overexpressed on target cells, are generally used in medicine. However, when the same antigen molecules (or similar ones in the context of antibody recognition) are expressed on different cells, e.g., on cell 1 and cell 2, these cells cannot be differentiated. Even if levels of antigen expression differ, strong interaction with the antibody results in low specificity (cells 1 and 2 in Figure 2a). As a result, to target a certain cell, we need to identify specific substances that are preferentially overexpressed on only this cell. Unfortunately, such overexpression is uncommon, especially in the human body. In most cases, various cells are covered by the same or similar molecules. This is a general limitation of using strong interactions in cell targeting.

Alternatively, single-glycan–protein interaction is very weak (mM–μM K_D_). However, when there are several glycan fragments in the cluster that can interact with cell lectins, the strength of interaction substantially increases. This effect is described as a multivalency or cluster effect. For example, both cells 1 and 2 in Figure 2b can interact with glycocluster; however, only cell 1 will be identified due to the multivalency effect (one glycan–lectin interaction with cell 2 will not cause the fixation).

**Figure 3:**
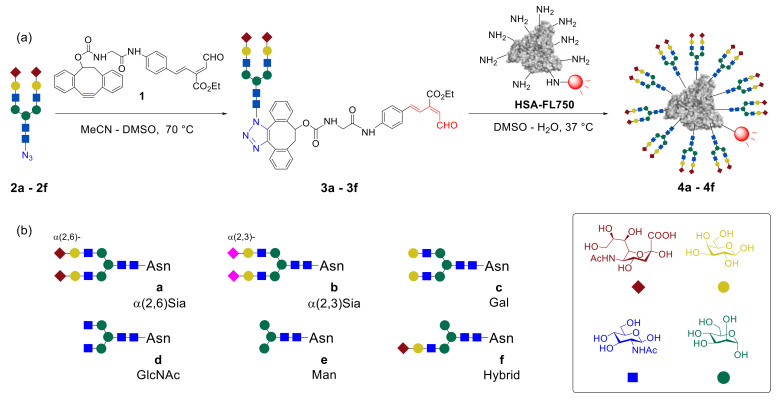
(**a**) Synthesis of homogeneous *N*-glycoalbumins **4a**–**f** using one-pot, double-click strategy; (**b**) structures of biantennary glycans **a**–**f** [43].

In the early stages of research, Tanaka et al. focused on the synthesis and biological evaluation of glycoalbumins, containing one type of biantennary glycan units—homogeneous *N*-glycoconjugates [43]. It was demonstrated that the usage of one-pot, double-click methodology allows efficient immobilization of a dozen *N*-glycans on albumin. Figure 3 shows a schematic representation of monosaccharides combined in *N*-glycans **a**–**f**. The *N*-glycan-azides **2a**–**f** were involved in the reaction with the unsaturated aldehyde **1** linker to yield glycan–aldehydes **3a**–**f**, which afterward reacted with HiLyte Fluor 750^®^-labeled human serum albumin (**HSA–FL750**), to afford the homogeneous glycoalbumins **4a**–**f** [43,45]. It should be noted that HSA was chosen as a template because of several important advantages such as the availability of more than 30 lysines for clusterization, serum stability, and insignificant immunogenicity in clinical utility.

Noninvasive imaging of the homogeneous glycoproteins clearly demonstrated the *N*-glycan-trimming dependence of in vivo kinetics and organ-specific accumulation of glycoalbumins, due to glycan multivalency effects. Thus, for example, the introduction of α(2,6)- and α(2,3)-disialoglycans increased the serum stability, compared with that of intact albumin. Glycoclusters **4a** and **4b** were cleared through the urinary bladder over 24 h. Trimming of the sialic acids fragments resulted in the shift of the glycoalbumin **4c** excretion through the gall bladder and the intestine. GlcNAc-terminated, mannose-terminated, and hybrid-type glycoalbumins **4d–f** did not exhibit preferential excretion properties but instead strongly accumulated in the liver [43].

Tanaka et al. also studied the tumor-targeting properties of homogeneous *N*-glycoalbumins **4a**–**f** [45]. Out of all the clusters examined, only α(2,3)-disialo-congener **4b** clearly visualized A431 tumors implanted in nude mice within 1 h. This glycocluster was then smoothly washed out of the tumor and excreted via the urinary bladder. These results showed glycoclusters as promising diagnostic tracers for use due to the good detection properties and rapid serum clearance.

According to the data presented above, glycoclusters are well established as various cells detection and visualization systems including tumor ones. However, the problem of glycoclusters usage as drug delivery systems is still a challenge. Tanaka et al. investigated their therapeutic in vivo synthetic chemistry strategy as a method for the synthesis of bioactive compounds in live animals [46]. According to this strategy, an inactive compound can be injected into the animal model and spread all over the body. This compound will react with cell surface reactive groups just in the presence of a catalyst. In this case, the catalyst delivery system is necessary, in order to supply the catalyst to the target cell/organ.

**Figure 4:**
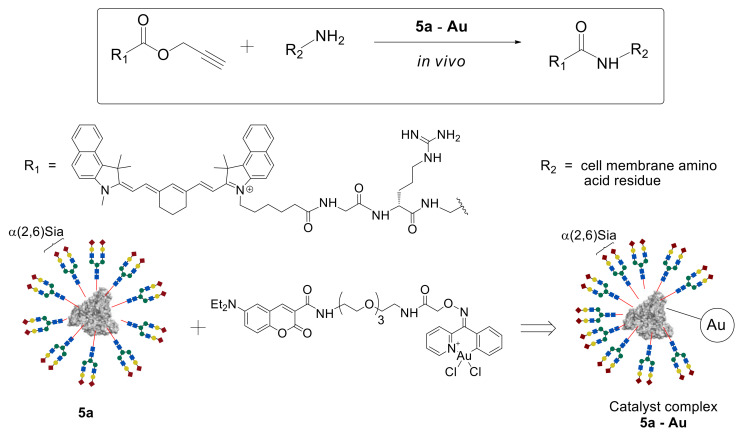
Reaction between fluorescent propargyl ester and amino groups of the surface cell membrane catalyzed by the homogeneous glycoalbumin based **5a**–**Au** complex.

In this study, propargyl ester of substituted acetic acid was used as an inactive starting compound, coumarin-containing Au (III) complex as the catalyst, and homogeneous glycoalbumin **5a** possessing α(2,6)Sia-terminated *N*-glycans as the catalyst delivery system (Figure 4). A metal-catalyzed reaction was explored between propargyl ester and amino groups of the protein, leading to the formation of an amide bond that could be carried out in the live animal. To perform the reaction in vivo, catalytic complexes **5a–Au** and **5c–Au** were synthesized based on homogeneous clusters **5a** and **5c** and coumarin-containing Au (III) complex [46].

The resulting **5a**–**Au** complex was injected into the BALB/cAJcl-nu/nu mouse model. Due to the presence of the α(2,6)Sia-terminated glycan **a** in the **5a**–**Au** complex, its targeted delivery to the liver cells was observed. After 30 min, a fluorescently labeled propargyl ester probe was injected into the mouse, and its biodistribution was studied by noninvasive fluorescence imaging. As expected, high fluorescence intensity was observed precisely in the liver cells, where the amide bond formation reaction between the ester and the cell surface membrane amino groups proceeded due to the accumulated **5a**–**Au** catalyst complex. A similar result was achieved by using the **5c**–**Au** system on the basis of Gal glycan **c** [46], which, as shown earlier [43], selectively accumulates in intestinal cells.

Thus, due to the use of targeted delivery of *N*-glycans and HSA-based glycoclusters, the possibility to carry out the metal-catalyzed, organ-specific amidation reaction in the mouse model was demonstrated for the first time. These results open up prospects for the synthesis of biologically active compounds directly in a living organism.

## 3. Arbitrarily Arranged Heterogeneous Glycoalbumins

**Figure 5:**
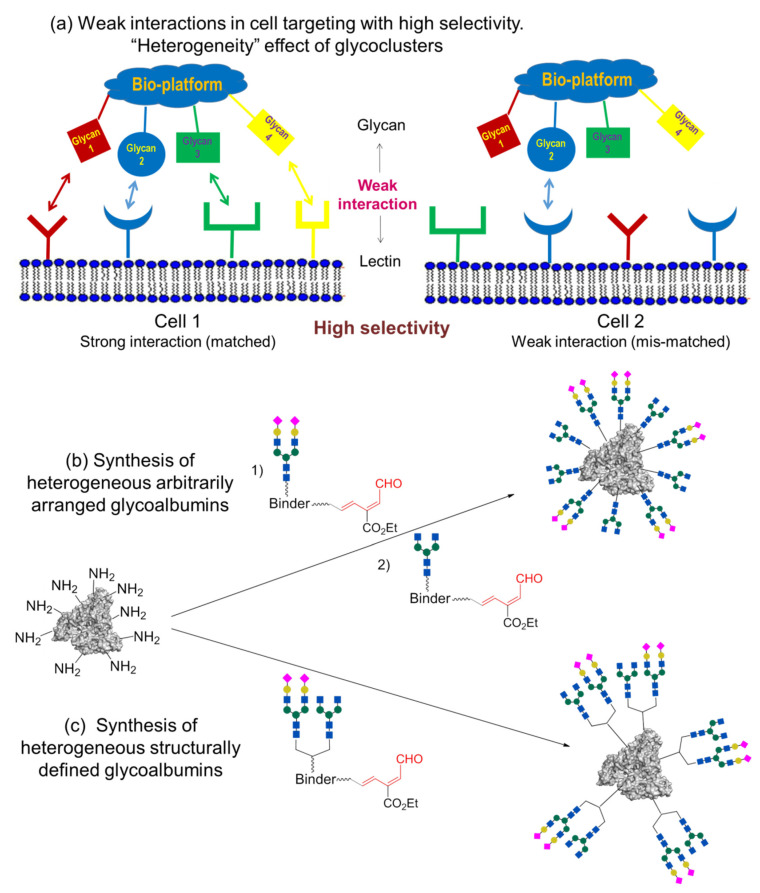
(**a**) Selective and strong interaction of heterogeneous glycoconjugates with target cells through pattern recognition using effect of heterogeneity. Glycans and lectins of the same shape and color interact with each other. Bio-platform: cell, antibody, protein, etc. Synthesis of two types of heterogeneous *N*-glycoalbumins containing two different *N*-glycan fragments—(**b**) arbitrarily arranged glycoalbumins and (**c**) structurally defined glycoalbumins [44]. Copyright Wiley-VCH GmbH. Reproduced with permission.

The pattern recognition concept described in this review is shown in Figure 2, which explains the effect of multivalency in cell recognition. However, natural glycoclusters do not consist of a single glycan structure (such as the homogeneous glycoclusters shown in Figure 2b) but rather mixtures of various structures, i.e., heterogeneous glycoclusters in Figure 5.

Glycoclusters containing different *N*-glycan fragments show high binding affinity to target cell 1, due to multiple glycan–lectin interactions (“matched” multivalency combination). Contrariwise, just a few glycan–lectin interactions (viewed as weak interactions) are available for the interaction of this glycoconjugate with other cells (shown in blue for cell 2, Figure 5a). The disposition of lectins on the surface does not correspond with the glycans’ arrangement (“mismatched” combinations). In consequence of such multiple glycan–lectin pattern recognition, the heterogeneous glycocluster can be involved in selective targeting of cell 1.

Although the studied homogeneous glycoclusters **4a**–**f** showed recognition of various cells, including cancer cells, selectivity was not high [43,45]. Greater natural macromolecule imitation can be achieved with the use of heterogeneous glycoconjugates carrying residues of several different glycans. The simplest models of heterogeneous objects are glycoclusters containing fragments of two different biantennary glycans.

As a further step, the RIKEN click strategy was applied to the synthesis of heterogeneous glycoconjugates [43,44,47,48]. Tanaka et al. developed two approaches, which are based either on the sequential introduction of the first, followed by the second, glycan into the protein molecule (Figure 5b, so-called arbitrarily arranged or positionally uncontrolled heterogeneous glycoalbumins), or on the initial incorporation of two different glycans into a single structural fragment, followed by its immobilization on the protein (Figure 5c, structurally well-defined heterogeneous glycoalbumins).

**Figure 6:**
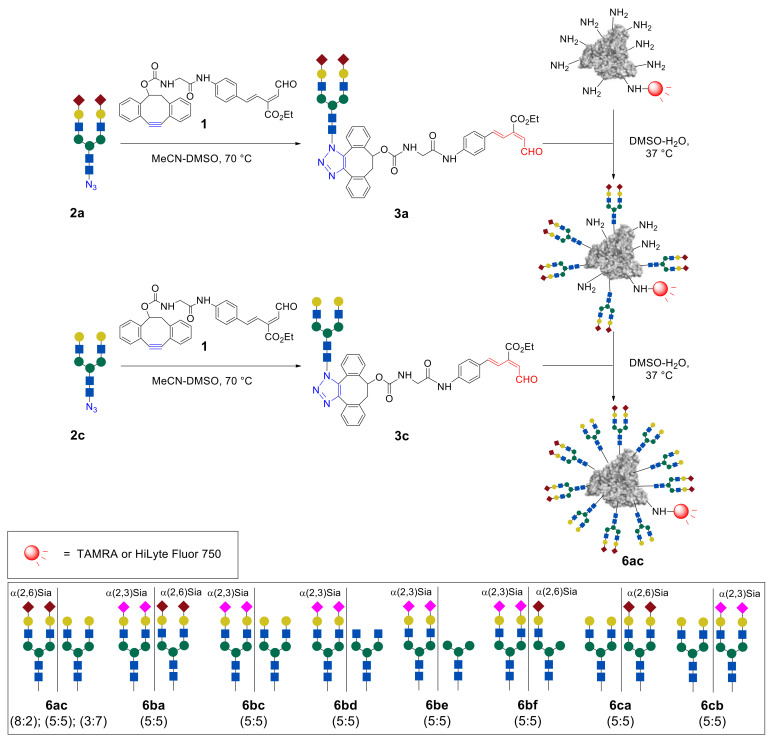
Synthesis of heterogeneous arbitrarily arranged glycoalbumins **6** by sequential immobilization of glycan–aldehydes **3**: representative synthesis of heterogeneous glycoalbumin **6ac**; synthesized glycoalbumins **6** (numbers in parentheses represent the ratios and order of the introduced glycans) [43].

Sequential immobilization of sialo- and asialoglycans **a** and **c** allowed the attainment of heterogeneous glycoconjugates **6ac** containing two different *N*-glycan fragments in different ratios [43] (Figure 6). Thus, at first, intermediate glycoconjugate possessing eight molecules of the sialo-*N*-glycan was obtained by reacting of **HSA**–**FL750** with 17.5 eq of the aldehyde **3a**. Then, immobilization of gal-*N*-glycan was achieved by the treatment of an intermediate cluster with 7.5 eq of aldehyde **3c**. The resulting heterogeneous glycoalbumin **6ac** contained sialo- and asialoglycans **a** and **c** with an 8:2 ratio. Clusters with other ratios of **a** and **c** glycans (**a**:**c** = 5:5 and 3:7) were also synthesized by the authors, by changing the equivalents of the glycan–aldehyde probes. Additionally, the inverse glycoalbumin **6ca** was similarly obtained by reacting of **HSA**–**FL750** first with asialo glycan–aldehyde **3c** and then with sialo glycan–aldehyde **3a**. The cluster with glycans ratio **c**:**a** = 5:5 was used in the studies of positional effects of glycans on biotemplate.

According to biodistribution studies, a change in the excretion pathway from gall bladder/intestine to the urinary bladder, depending on the number of sialo-*N*-glycans on proteins **4a**,**c** and **6ac**, was observed [43]. Specifically, the introduction of nonreducing end sialic acids on albumins induced preferential urinary bladder excretion. It is important to be mentioned that a comparison of glycoalbumin **6ac** with inverse glycoalbumin **6ca** showed more rapid and complete intestinal excretion of **6ca**. Thus, the positions of immobilized glycans, together with the ratio of the glycans on the cluster, are very essential factors for inducing the effects of heterogeneity.

**Figure 7:**
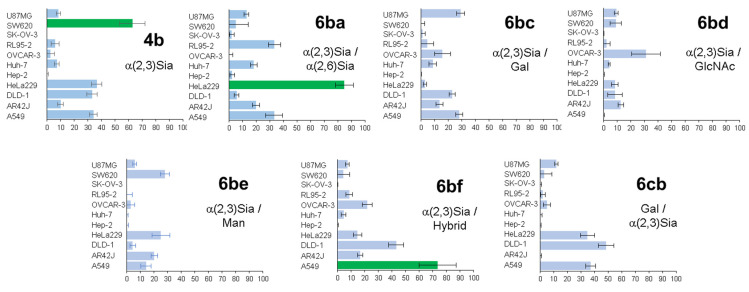
Study of homogeneous glycoalbumin **4b** and heterogeneous glycoalbumins **6ba**–**cb** (100 nM) against 11 different cell lines performed with cell-based assay via fluorescence: green bars—strong binding; blue bars—moderate or negligible binding. Reproduced from [47], with permission from the Royal Society of Chemistry.

The next stage consisted of the study of heterogeneous glycoclusters for selective detection of various cancer cells [47]. Different heterogeneous glycoalbumins (Figure 6 and Figure 7) were synthesized with the use of combinations of five different glycan assemblies (α(2,3)Sia-, α(2,6)Sia-, Gal-, Man-, and GlcNAc-terminated glycans). For cell-based studies, glycoalbumins **6** were labeled with the fluorescent TAMRA dye. Notably, heterogeneous glycoalbumin **6cb** exists as the “regioisomer” of glycocluster **6bc**, where the positions of conjugated α(2,3)Sia- and Gal-terminated glycans are switched.

Heterogeneous glycoalbumins **6ba**–**cb** were incubated with 11 different cell lines and then imaged to determine the extent of cell binding via fluorescence (Figure 7). The results obtained from biological experiments revealed three main trends in relation to cancer cell binding [47]. First, it was shown that even small changes to introduce heterogeneity can lead to significant changes in pattern-recognition-based binding. Thus, for example, despite the fact that the only structural difference between compounds **4b** and **6ba** is the nature of the link between the sialic acid and galactose fragments, glycoclusters show various binding affinities to cancer cell lines. The homogeneous glycocluster **4b** is characterized by strong binding with SW620 cells, while the heterogeneous conjugate **6ba** shows preferential cell binding with cancer HeLa cells.

Secondly, in the case of sialic acid-containing glycoalbumins, the total negative charge of a cluster has a great influence on binding efficiency. Comparison of fluorescence microscopy data showed that glycoalbumins with a larger number of sialic acid fragments yield stronger interactions.

The third observed trend is that positioning of the glycan units is also an important factor for pattern recognition. Thus, although the glycan constitution of both **6bc** and **6cb** is made of α(2,3)Sia- and Gal-terminated glycans, the pattern of their interaction with various cancer cell lines is significantly different (Figure 7) [47].

Glycoalbumins **6ba**, **6bc**, and **6bd**, labeled with a near-infrared fluorophore, HiLyte Fluor 750^®^ were also synthesized, in order to screen these clusters for in vivo tumor tissue targeting [47]. Data analysis showed that, in general, all glycoalbumins were subjected to the conventional excretion pathway typical for proteins such as serum albumin. Following distribution around the body, glycoalbumins were digested in the liver, trafficked to the kidneys, and then urinary bladder, to be excreted from the body. The study revealed glycoalbumin **6ba**, which selectively and strongly binds to HeLa229 tumor cells in live mice at a dose of 1.5 nmol/100 μL.

It is important to note that the positions of HSA where glycans react could be controlled. A multipronged approach based on the joint use of LC–MS/MS and MALDI–TOF/MS mass spectrometry methods made it possible to identify the preferential lysine fragments subjected to ligation. According to the mass spectrometry data analysis, the lysine groups in positions 195, 199, 525, 536, and 541 are the most reactive and preferred ones for the interaction with glycan–aldehydes [47].

**Figure 8:**
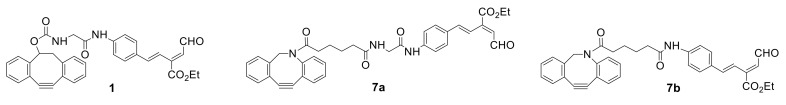
Structures of unsaturated aldehydes **1** and **7a** and **7b**, containing strained triple bond for glycans immobilization on albumin via one-pot, double-click strategy.

As it was shown above, the two-stage method developed for the synthesis of glycoproteins is very convenient and effective. However, the synthesis of the key intermediate **1** containing dibenzocyclooctyne and unsaturated aldehyde fragments (Figure 1) is very complicated and time consuming [38]. In their research, Tanaka et al. always seek to improve the synthetic methodology [49,50]. Thus, at first, it was suggested to replace compound **1** with the similar unsaturated aldehyde **7a** (Figure 8), which can be easily obtained from the commercially available carboxylic acid in three steps [49]. It was shown that the new key intermediate **7a** is more reactive in the terms of both click reactions and could be successfully used in the synthesis of various glycoalbumins [44]. Moreover, in order to further enhance their methodology, researchers optimized the synthesis of starting compound that responds to the unsaturated aldehyde fragment and represented improved key compound **7b** [50,51]. It was shown that the linkage between *N*-glycans/aldehyde and proteins did not alter the RIKEN click reactivity or cell interaction profiles. Once the aldehyde probe could be easily and reproducibly accessed at a large scale, it could be useful for glycoconjugations and the development of glycocluster-based drug delivery systems.

## 4. Structurally Well-Defined Heterogeneous Glycoalbumins

As an alternative, Tanaka et al. have also developed more structurally defined heterogeneous glycoalbumins, by simultaneously introducing two different *N*-glycans that were linked in advance by the RIKEN click reaction (Figure 6c) [44,48]. This glycoconjugation method is superior in terms of the controlled spatial arrangement of two glycan molecules relative to one another.

**Figure 9:**
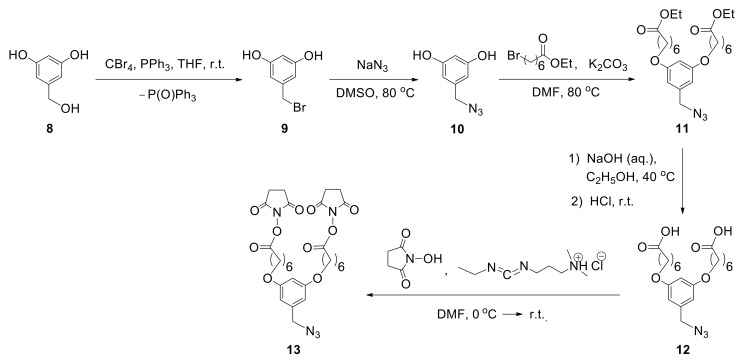
The sequence of transformations of 3,5-dihydroxybenzyl alcohol (**8**) to target azide **13** [48].

According to the proposed method, to incorporate two biantennary glycans in one unit, the design and multistep synthesis of an azide precursor were carried out. Azide **13** was chosen as a precursor, containing two branches with NHS groups required for the introduction of various glycans, and an azide group used for subsequent conjugation with albumin (Figure 9). The use of commercially available 3,5-dihydroxybenzyl alcohol (**8**) as the starting compound allowed the introduction of azide group by sequential substitution reactions OH—Br—N_3_. Then, two phenolic hydroxyl groups were transformed to the target compound **13** by alkylation reaction, with the subsequent hydrolysis and activation of carboxyl functions [48].

**Figure 10:**
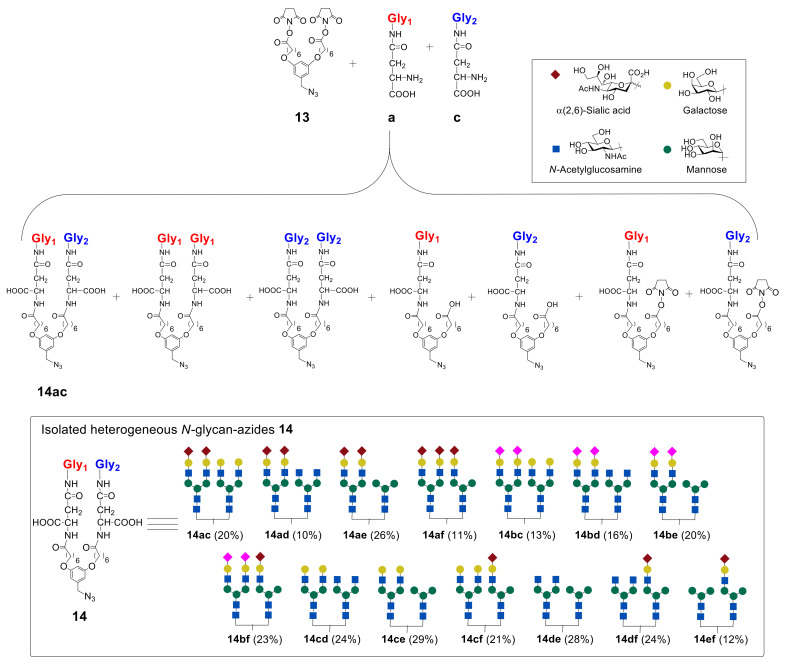
Reaction of azide **13** with two different *N*-glycans leading to the formation of heterogeneous glycan–azides **14ac**–**ef**, represented by the synthesis of **14ac** [44]. Copyright Wiley-VCH GmbH. Reproduced with permission.

In the next step, the authors synthesized heterogeneous *N*-glycan-azides **14ac**–**ef** in a series of reactions (Figure 10). It was found that the best result in terms of the yield of heterogeneous glycan–azides **14ac**–**ef** can be achieved by carrying out the reaction in DMF in the presence of diisopropylethylamine (*i*Pr_2_EtN), with the initial addition of glycan with a higher molecular weight into the reaction. It was also revealed that the reactivity of glycans toward the starting azide **13** decreases in the following order: Man ˃ GlcNAc ˃ Hybrid ≥ Gal ˃ α(2,3)Sia ≈ α(2,6)Sia [48].

**Figure 11:**
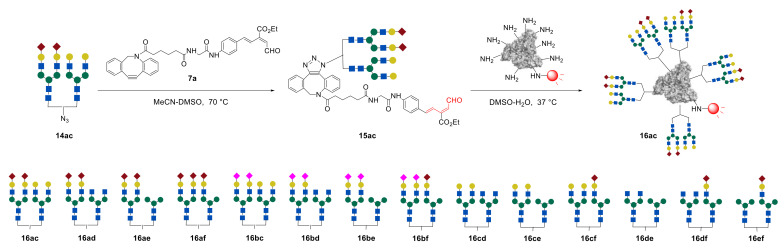
Synthesis of heterogeneous structurally well-defined glycoalbumins **16** containing two different *N*-glycan fragments by simultaneous immobilization of heterogeneous glycan–aldehydes **15**: representative synthesis of heterogeneous glycoalbumin **16ac**; synthesized glycoalbumins **16ac**–**ef** [44]. Copyright Wiley-VCH GmbH. Reproduced with permission.

Synthesized glycan–azides **14ac**–**ef**, containing two different *N*-glycans fragments were further immobilized on albumin via RIKEN click reaction, as discussed above. Notably, by varying the concentrations of the glycan–aldehydes **15**, different numbers of glycan units can be immobilized on albumin. Thus, heterogeneous glycoclusters **16ac**–**ef**, containing on average 10 or 4 *N*-glycan fragments on albumin were obtained (Figure 11) [44,48].

The study of the main biodistribution properties of clusters **16ac**–**ef** revealed that structurally well-defined heterogeneous glycoalbumins **16ac**, containing α(2,6)-sialic acid/galactose-terminated glycans, exhibited different excretion properties. This depends on the glycans’ spatial heterogeneity, resulting in rapid translocalization from the gallbladder to the intestine, even when compared with homogeneous Gal-terminated clusters **4c**. Previously, it was shown that homogeneous hybrid-type glycoalbumin **4f** selectively accumulated in the liver. Meanwhile, similar heterogeneous structurally well-defined α(2,6)-Sia/Man-terminated glycoalbumin **16ae** was selectively excreted through the urinary bladder [48]. Thus, even small amounts of glycans immobilized onto albumin were sufficient to produce multivalency and heterogeneity effects in the gallbladder/intestine translocalization properties in the context of structural heterogeneity. The obtained in vivo data showed that, in addition to the number of glycan fragments immobilized on albumin, the spatial arrangement of *N*-glycans in the heterogeneous glycocluster also affects the ability of *N*-glycoalbumins in excretion and organ-specific accumulation in vivo, due to the pattern recognition mechanism of glycans.

**Figure 12:**
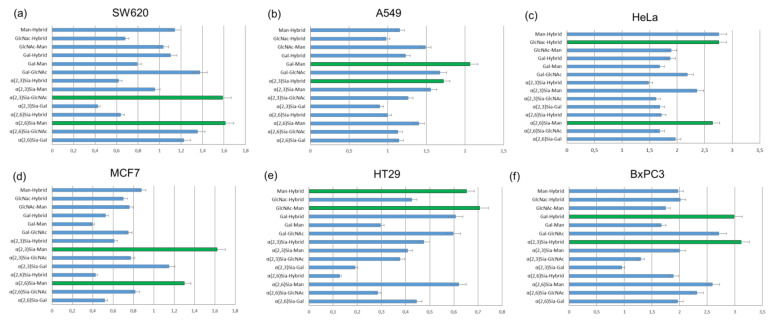
To screen binding affinities, 6 various cancer cell lines were incubated with 14 heterogeneous glycoconjugates **16ac**–**ef**. The strongest glycoalbumin–cell interactions are colored by green bars. Fluorescence was averaged from 10,000 cells and normalized (*n* = 4) [44]. Copyright Wiley-VCH GmbH. Reproduced with permission.

The results of cell-based experiments summarized in Figure 12 indicate that depending on the monosaccharides fragments united in the *N*-glycans, heterogeneous glycoalbumins **16ac–ef** show various binding affinities to different cell lines [44]. Therefore, for example, the combination of galactose- and mannose-terminated glycans **c** and **e** in glycocluster **16ce** results in strong interaction with A549 cells (Figure 12b), with almost no interaction with MCF7 cells (Figure 12d). Another important point to be mentioned is that glycan microheterogeneity had a large influence on pattern recognition mechanisms when two different *N*-glycans were combined in one unit. This could be exemplified by BxPC3 cells. Glycocluster **16bf**, carrying α(2,3)Sia-terminated glycan **b** and hybrid glycan **f**, exhibits strong interaction toward BxPC3 cells. The screening data on glycoalbumin–cell surface lectins’ binding affinity, summarized in Figure 12, distinctively demonstrate the importance of glycan heterogeneity for pattern recognition of targeted cells, as it was proposed.

Tanaka et al. also investigated in vivo experiments with structurally well-defined heterogeneous glycoclusters [44,51]. According to the cell-based experiments (Figure 12a), SW620 cells showed strong interaction with glycoalbumin **16bd** (α(2,3)Sia/GlcNAc-terminated glycocluster) but rather weak interaction with glycoalbumin **16bc** (α(2,3)Sia/Gal-terminated glycocluster). The only difference between the clusters is the presence or absence of galactose residue. Biological experiments in animal models improved the hypothesis that even a small difference in glycan local heterogeneity, i.e., that of galactose and glucosamine, can significantly alter cancer-targeting efficiency, at both cell and animal levels. It was shown that glycoalbumin **16bd** can accumulate in SW620 cancer cells; meanwhile, no **16bc** glycocluster can be found in the same tumor cells [51]. Such a pattern recognition property of heterogeneous glycoclusters is highlighted and differentiated from the other targeting methods, enabling the precise tuning of the interaction ‘‘on-demand” for the various targets in vivo.

**Figure 13:**
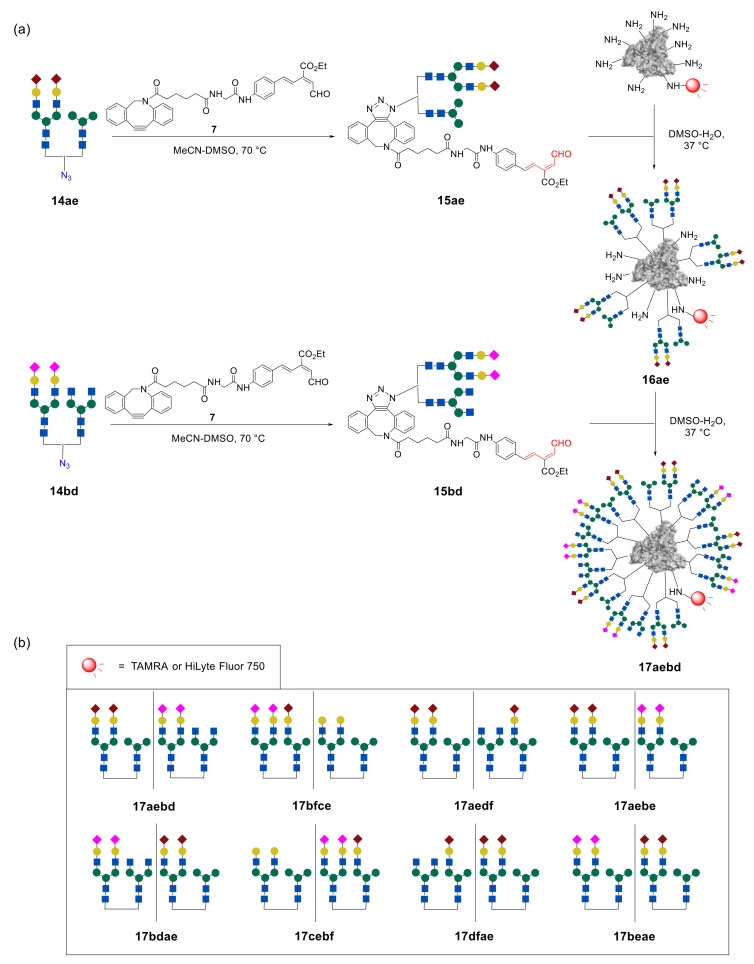
Immobilization of four different biantennary *N*-glycans on albumin in the synthesis of heterogeneous glycoclusters with the use of two heterogeneous glycan–aldehydes **15**: (**a**) representative immobilization of glycan–azides **14ae** and **14bd** onto albumin resulting in the glycoconjugate **17aebd**; (**b**) the range of synthesized glycoalbumins **17** [44]. Copyright Wiley-VCH GmbH. Reproduced with permission.

In order to increase the selectivity of glycan–lectin interaction, Tanaka et al. further investigated a new strategy for targeting the cancer cells by using higher-order glycan pattern recognition, e.g., glycoclusters carrying four different glycan moieties [44]. To combine four different glycan units into one cluster, two different *N*-glycans in one glycan–azide fragment were initially introduced. Then, sequential immobilization of this type of glycan–azide on albumin resulted in the formation of targeted higher-order glycoconjugates. A representative example is shown in Figure 13. Initially, about five fragments of heterogeneous glycan–aldehydes **15ae** were immobilized on fluorescently labeled albumin, to form intermediate glycocluster **16ae**. Thereafter, heterogeneous glycan–aldehyde **15bd** was attached to the intermediate glycoalbumin **16ae**. As a result, the higher-order heterogeneous glycoalbumin **17aebd**, containing four different *N*-glycan units with an average of about 20 oligosaccharide fragments, was synthesized.

The glycan structures to be immobilized on albumin were chosen on the basis of cell-based experiments of lower-order glycoclusters (Figure 12). Thus, for example, cluster **17aebd** is a combination of glycoalbumins **16ae** and **16bd**, which showed the highest selectivity for the target cells SW620. It was proposed that, in this case, glycans can multiply the interaction, to more selectively target the cancer cells.

Tanaka et al. synthesized four different glycoclusters—**17aebd**, **17bfce**, **17aedf**, **17aebe**—for targeting SW620, HeLa, A549, or MCF7 cell lines [44]. Due to the fact that pattern recognition is very sensitive to the spatial arrangement of glycans, the authors also prepared four glycoclusters—**17bdae**, **17cebf**, **17dfae**, **17beae**—that can be termed as “regioisomers” of clusters **17aebd**, **17bfce**, **17aedf**, **17aebe**, by changing the sequence of glycans immobilization on HSA.

**Figure 14:**
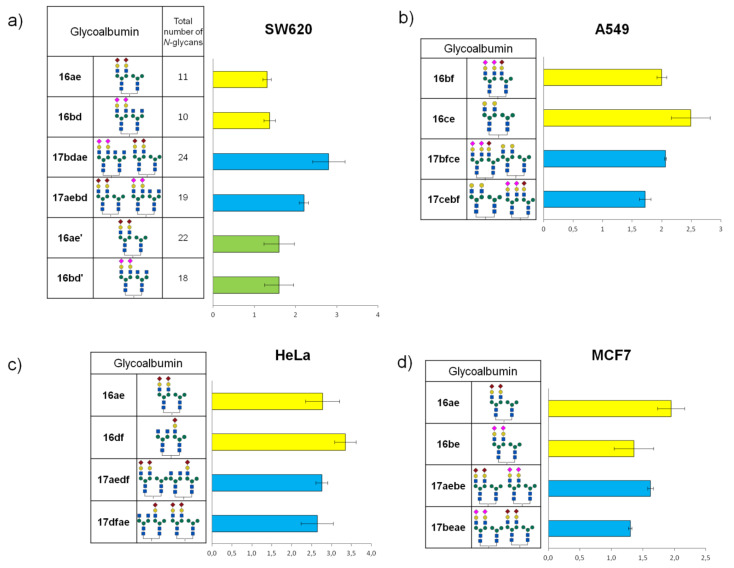
Heterogeneous glycoalbumins **17** containing four *N*-glycan moieties were incubated with corresponding cancer cell lines (**a**) SW620, (**b**) A549, (**c**) HeLa, and (**d**) MCF7, and the binding efficacy was compared with the same of glycoclusters **16**, carrying two different glycan fragments. Bars colored with yellow show fluorescence intensity of cells incubated with heterogeneous glycoalbumins **16** with two glycan fragments; blue bars—heterogeneous glycoalbumins **17** with four glycan fragments; green bars—glycoalbumins **16′** containing about 20 glycan molecules. Fluorescence was averaged from 10,000 cells and normalized (*n* = 4) [44]. Copyright Wiley-VCH GmbH. Reproduced with permission.

According to the cell-based experiments of each higher-order heterogeneous glycoalbumin pair, one or both regioisomers **17** demonstrated slightly but distinctly higher-targeting efficiency, compared with the initial, lower-order heterogeneous glycoclusters **17**, containing only two glycan fragments (Figure 14). It was shown that heterogeneity, but not multivalency effects, makes the major contribution to improving the targeting efficiency of higher-order heterogeneous glycoalbumins **17** [44].

It is important to mention that the degree of interaction shown in Figure 14 was diverse for different cell lines (HeLa, A549, MCF7, and SW620). Hence, the data intelligibly demonstrate that combining the four different glycan units within one high-ordered glycocluster, caused matched or mismatched pattern recognition to each cell line. This is the main underlying mechanism in glycan pattern recognition, as we presented in Figure 5a, which was most emphasized in this research.

Relying on cell-based analysis data shown in Figure 14, Tanaka et al. finally performed a preliminary study of in vivo cancer targeting by lower- and higher-order glycoalbumins [44]. Lower-order clusters **16ae** and **16bd** showed good agreement with cell interaction data. On the other hand, glycoconjugate **17aebd** showed high binding affinity to SW620 cancer cells in cell-based experiments, but barely detectable fluorescence was registered for SW620 cancer tissues. According to the time-dependent, whole-body imaging, glycoalbumin **17aebd** was rapidly excreted after injection. It is a general phenomenon that the larger the molecular weight of molecules is, the faster the excretion occurs. In this study, the molecular weight of glycocluster **17aebd** is two times more than **17ae**, which might alter the pharmacokinetics of albumin. In this respect, in the development of heterogeneous glycoclusters as new drug delivery systems, the in vivo stability and kinetics of clusters should be taken into consideration as the main feature. This is a great challenge in the field of molecular imaging and is not a trivial problem to solve. However, the preliminary screening on hepatocytes in vitro may not result in any essential enhancement.

## 5. Conclusions

Glycans exist in nature as simple and complex carbohydrates and are involved in various processes of living systems. Although glycans are attached to almost all membrane proteins and have diverse functions, the molecular basis of glycan functions remains to be elucidated. The major features of glycans in pattern recognition are their multivalency and heterogeneity. Today, significant advances have been made in protein glycoengineering as an important way to improve the performance of therapeutic proteins and industrial enzymes. However, this technology is still in its infancy and has little acceptance in the scientific community.

In this regard, the synthesis of glycoproteins of a certain structure has considerable prospects and has been widely studied in recent decades. In this review, we briefly mentioned some strategies for the synthesis of glycoclusters on albumins and their biological evaluation. The main focus was on the conventional method for the synthesis of glycoalbumins via one-pot, double-click methodology, including strain-promoted click reaction, followed by the subsequent 6π-azaelectrocyclization of unsaturated imines (RIKEN click reaction). This methodology was successfully proven in the synthesis of various *N*-glycoconjugates—homogeneous and heterogeneous. It should be noted that heterogeneous glycoalbumins are structurally defined and can be obtained by two different protocols; meanwhile, their combination allows the attainment of heterogeneous clusters containing up to four different glycan moieties.

The characterization of the in vitro and in vivo biological behavior of various types of glycoclusters sheds light on the importance of the glycoconjugates’ multivalency and heterogeneity effects in pattern recognition mechanisms. It can be concluded that the structure of glycan moiety, glycan valency on albumin, positions of glycans within the molecules, and their arrangement relative to one another are all factors very significant in highly effective glycan–lectin interaction.

Fast and selective accumulation of *N*-glycoconjugates in specific tissues and organs in vivo renders this class of compounds as extremely promising targeted delivery systems. It is expected that such a strategy, once fully established, should greatly promote the advancement of glycomics research. Using the features inherent in lectin–glycan interactions may lead to the creation of a new class of tracers based on *N*-glycans for the detection and visualization of target tissues and organs, as well as the treatment of various diseases.
